# Collective problem-solving in Japanese primary mathematics lessons

**DOI:** 10.1007/s10649-025-10400-5

**Published:** 2025-04-12

**Authors:** Valérie Batteau, Takeshi Miyakawa, Minbom Ryu

**Affiliations:** 1 UER MS – Lausanne Laboratory Lesson Study, Lausanne University of Teacher Education, Lausanne, Switzerland; 2 Waseda University, Tokyo, Japan; 3 Korea University, Tokyo, Japan

**Keywords:** Japanese mathematics teaching, Structured problem-solving lesson, *Milieu*

## Abstract

This study investigates the characteristics of Japanese primary school mathematics lessons that adopt a problem-solving approach. We argue that these characteristics are reflected in three key aspects: collective teaching and learning, the lesson as a “drama” (i.e., its structured flow), and the focus on mathematical knowledge. In this paper, we refer to these aspects as the collective dimension, chronological dimension, and epistemological dimension of Japanese mathematics lessons, respectively. Using the Anthropological Theory of the Didactic (ATD) as a theoretical framework, we analyze the mathematics lessons of a Japanese primary school, focusing on the evolution of *milieus* and the three geneses of mathematical knowledge (*topogenesis*, *chronogenesis*, and *mesogenesis*). Our aim is to highlight these dimensions and further identify the distinct features of Japanese lessons, as well as to demonstrate how, and to what extent, the evolution of *milieus* effectively captures the structure and dynamics of the Japanese lessons.

## Introduction

In recent decades, Japanese mathematics teaching and teacher professional development have garnered particular attention from mathematics educators worldwide, especially thanks to the TIMSS Video Study, an international comparative study of mathematics lessons (Stigler & Hiebert, 1999). One of the key aspects that has drawn international interest is that mathematics teaching in Japanese schools frequently aligns with a problem-solving approach, referred to in Japanese as *mondaikaiketsu-gata jugyō* and in English as *structured problem-solving lessons* (Stigler & Hiebert, 1999). In this approach, a lesson is typically organized with a single problem, with the teacher orchestrating the class to develop mathematical thinking and promote students’ active and autonomous engagement. This characteristic has been extensively documented in both English (Hino, 2007, 2015; Nunokawa, 2015; Takahashi, 2006, 2008) and French (Batteau & Miyakawa, 2020) literature on mathematics education.

A typical Japanese structured problem-solving lesson is often characterized by the following phases: *hatsumon*, *kikan-shidō*, *neriage*, and *matome* (see Sect. 2.1). Several studies have investigated the structure of lessons comprising these phases and the teacher’s role in each phase (Clarke et al., 2006; Stigler & Hiebert, 1999). For example, the *neriage* phase, where the teacher manages students’ different ideas and develops mathematical concepts and methods in a whole-class setting, has received particular attention as a key phase in the success of problem-solving lessons (e.g., Groves & Fujii, 2008; Inoue, 2011; Takahashi, 2008).

One important characteristic of Japanese problem-solving lessons, which we seek to highlight, is their collective dimension. Here, the term *collective* does not refer to students working collectively in group, but rather to the dynamic classroom interactions involving both students and the teacher. A prominent example of this is the *neriage* phase mentioned above. In fact, in many European educational contexts, such as Switzerland, teaching often places greater emphasis on individual learning (Clivaz & Miyakawa, 2020).

While previous studies have investigated the structure of Japanese lessons and the functions of each phase (Clarke et al., 2006; Shimizu, 2006), the collective dimension has not yet been fully explored. The collective dimension appears, in our view, not only in some specific phases, such as *neriage*, but throughout the lesson. Funahashi and Hino (2014) indirectly refer to this dimension through the concept of the “guided focusing pattern” from the perspective of social interaction patterns in classrooms. This pattern describes the interactive process through which the teacher guides classroom discussions to elicit mathematical ideas and focus on new mathematical content. In line with this study, we are interested in uncovering how different ideas developed individually or in groups by students contribute to the collective development of mathematical ideas in the classroom. While previous empirical studies have identified interaction patterns, our goal in this paper is to characterize the process of such development.

This research is based on *the Anthropological Theory of the Didactic* (Chevallard, 2019; Chevallard & Bosch, 2020), a theoretical framework of French origin. Specifically, we employ the concept of *milieu* to examine the distinctive characteristics of Japanese problem-solving lessons. The aim of this paper is, therefore, to highlight the distinct features of Japanese mathematics lessons and to illustrate how, and to what extent, the evolution of *milieus* can serve as an analytical lens to identify these characteristics through the analysis of concrete Japanese lessons.

In what follows, we first discuss the context of mathematics teaching in Japan and review previous studies on problem-solving lessons (Sect. 2) and then introduce the concept of *milieu* and its theoretical foundations (Sect. 3). Section 4 is devoted to an analysis of Japanese primary mathematics lessons, exemplifying how and to what extent the characteristics of these lessons can be identified.

## Mathematics teaching in Japan

This section presents a general overview of Japanese mathematics lessons based on a review of the English literature. The first part introduces the structured problem-solving lesson and shows the importance of the collective dimension, the second part concerns the importance of the flow of the lesson or the chronological dimension, and the last part highlights the mathematical knowledge or the epistemological dimension on which the Japanese lesson focuses.

### Structured problem-solving lesson

In Japanese primary schools, ordinary mathematics lessons are often conducted as a form of structured problem-solving lesson (Batteau & Miyakawa, 2020; Fujii, 2018; Isoda, 2012; Isoda & Nakamura, 2010; Miyakawa & Winsløw, 2009; Shimizu, 1999, 2009b, 2015; Stigler & Hiebert, 1999; Takahashi, 2006, 2008). The structure of this form of lesson is often described by the following phases:

- *Hatsumon*: presentation of the problem of the day, eventually followed by the *mitōshi* (estimation, planning, or anticipation of the solution)
- *Kikan-shidō:* instruction at students’ desks. Individual student work, eventually followed by the group work
- *Neriage*: whole-class discussion on students’ ideas, strategies, and/or solutions
- *Matome*: summary by the teacher, eventually followed by further development or enhancement of the problem

One characteristic of the Japanese problem-solving lesson is its collective dimension. Three of these four phases—*hatsumon*, *neriage*, and *matome*—unfold collectively, and the teacher plays a crucial role. In the *hatsumon* phase, the teacher ensures that all students understand the problem through interactions with them, such as asking a conjecture about the problem and sharing it in the classroom. In the *neriage* phase, the teacher manages the classroom discussion to collectively construct the new knowledge or method and develop students’ mathematical thinking. During this collective phase, the class compares and discusses students’ ideas, strategies, and solutions. The teacher’s role is to orchestrate students’ ideas, to highlight important mathematical ideas to reach the goals of the lesson, and to help students polish their solutions to learn mathematical content (Shimizu, 1999). *Matome* is again a collective phase that serves to explicitly achieve the main goals of the lesson. In this phase, the teacher summarizes the activities of the lesson and institutionalizes the new knowledge or methods aimed at in the lesson.

The formalization of the structure of problem-solving lessons in Japan differs according to Japanese researchers (Fujii, 2018; Isoda, 2012; Isoda & Nakamura, 2010; Miyakawa & Winsløw, 2009; Shimizu, 2009b, 2015) and American researchers (Stigler & Hiebert, 1999), but all agree with the existence of different specific moments (phases or activities) in the lessons and its collective dimension as a characteristic of Japanese problem-solving lessons.

### Japanese lesson as a “drama”

Another characteristic of Japanese problem-solving lessons is the flow of the lesson. The lesson is sometimes considered a “drama” or story that includes a chronological order of activities from the introduction to the climax (Hirabayashi, 2002; Shimizu, 2009a).

The pattern and structure of Japanese problem-solving lessons have interested researchers, particularly in the context of international comparisons of mathematics teaching. The Learner’s Perspective Study was an international project that started in Australia, Germany, and Japan to report on national norms of teaching practices with an in-depth analysis of mathematics classrooms by focusing not on a single special lesson but on a unit of consecutive lessons in an ordinary setting (Clarke et al., 2006). This project highlighted a characteristic of Japanese problem-solving lessons that seems critical to understanding its collective dimension. By examining the structure of an exemplary Japanese lesson, Shimizu (2009a) emphasized this characteristic with the metaphor of a story or a drama derived from traditional Chinese poems. The lesson follows the structure of a story with four steps from the beginning (starting point), the development of the story, and the twist point of the story with an unforeseen event toward the end (summary of the whole story). For Japanese teachers, a lesson includes a twist point that usually occurs during the whole-classroom discussion (Shimizu, 2009a). Furthermore, in Japan, the lessons are not considered isolated but are connected to each other within a sequence of lessons, called *unit* (*tangen* in Japanese).

Taking into consideration this characteristic of Japanese lessons related to the flow of lessons, or lessons as a “drama,” it will be necessary to have a tool that allows us to analyze the collective development of mathematical ideas related to their chronological dimension throughout a whole lesson regardless of the phases.

### Focus on mathematical knowledge

As mentioned above, the structure and flow of the problem-solving lesson constitute a cultural characteristic. Another characteristic is the place allocated to mathematical knowledge in ordinary lessons. The collective teaching during the *neriage* phase serves to prepare the next phase, stating and summarizing the mathematical knowledge. Knowledge is placed at the center of teaching. This fact can be explained by the Japanese development of the problem-solving approach as an approach for learning mathematics rather than as a way to learn problem-solving skills (Clivaz & Miyakawa, 2020; Takahashi, 2008). In other words, Japanese teaching is linked to a specific goal of the lesson, which is the development and learning of new mathematical knowledge. This is a characteristic of the Japanese problem-solving lesson found in its epistemological dimension.

The analysis of the lesson by the evolution of *milieus* models the mathematical knowledge at stake and its chronological development in relation to the collective work, specifically how mathematical ideas are gradually developed during individual and collective work. In this respect, we adopt the ideas developed in the theoretical framework of French origin, which fully take into account the epistemological dimension of mathematics teaching (Artigue, 1990; Florensa et al., 2015; Gascón, 2003): *the Anthropological Theory of the Didactic* (ATD).

## Concept of *milieu* and its development

This section introduces the concept of *milieu* in ATD as a framework for understanding the development of mathematical knowledge in both collective and individual settings within Japanese problem-solving lessons. There are two concerns regarding the notion of *milieu*. First, we need to identify the elements of the *milieu* that chronologically evolve throughout the learning process. Second, we must characterize the collective development of mathematical knowledge by the teacher and students in a whole-class setting. To address the first concern, we adopt key ideas from ATD. For the second, we examine the individual, group, or collective interactions with the *milieu*, exploring how these interactions derive their evolution in the classroom.

The concept of *milieu* initially appears in ATD (Chevallard, 1992) as *institutional milieu*, referring to an institution and its institutional temporality rather than a specific *situation* (Perrin-Glorian, 1999), as is the case in the *Theory of Didactical Situations* (TDS) (Brousseau, 1997; Warfield, 2014). Unlike in TDS, where the *milieu* is formulated as a dynamic component of problem-solving situations, in ATD, it is initially conceptualized as a stable or transparent set of objects and their institutional relations at a given time for a person within the didactic system.

However, later theoretical developments in ATD placed the concept of *milieu* at the forefront, referring to it as a tool for describing mathematics learning. This is in the context of inquiry-based teaching and learning, called *Study and Research Paths* based on the paradigm of *questioning the world* (Chevallard, 2015). Within this theoretical development, the *milieu* has further evolved as a crucial concept to characterize the inquiry process undertaken by the learner.

Inquiry based on the paradigm of *questioning the world* has two main characteristics. First, a question serves as the primary driver of inquiry, with the investigation process yielding not only answers but also new questions. Second, inquiry involves searching for different information useful to the inquiry as a critical process, beyond understanding the given information and developing one’s own answer. Within ATD, these processes are characterized in terms of the *dialectic of media and milieu* (Chevallard, 2007; Chevallard & Bosch, 2019; Kidron et al., 2014). *Milieu* denotes here a system that can be regarded as devoid of intention in the answer it can make, explicitly or implicitly, to a particular question and acts as a fragment of “nature” (Chevallard, 2007). It is called *didactic milieu*. *Media*, in contrast, means any system that issues messages, any system of representation of a part of the natural or social world addressed to a certain public (Chevallard, 2007; Chevallard & Bosch, 2019).

The theoretical development related to inquiry further led to the formulation of the process of inquiry (or even the process of teaching and learning) in terms of *the Herbartian schema* (Chevallard & Bosch, 2019) and through the concept of *milieu*. Within ATD, the didactic system of inquiry is expressed as *S*(*X*, *Y*, *Q*), representing a group of persons *X* (e.g., students) studying a question *Q* with support from another group of persons *Y* (e.g., teachers). In the process of studying *Q*, the didactic system generates the *milieu M* and produces its own *answer *A^❤^, specific to *Q*, by working on *M*. This process is formalized as follows:

[S(X;Y;Q)➥M]➦*A*^❤^.

The *milieu M* can be specifically expressed as follows:

*M* = {*A*^⋄^_1_, *A*^⋄^_2_, …, *A*^⋄^_m_, *W*_m+1_, *W*_m+2_, …, *W*_n_, *Q*_n+1_, *Q*_n+1_, …, *Q*_p_, *D*_p+1_, *D*_p+2_, …, *D*_*q*_}.

It includes preexisting *answers A*^⋄^¸ students may find from the *media*, different *works W*, used to make sense of and analyze the answers and to construct answers, *questions Q* induced by other elements (e.g., answers and works) or raised by the construction of the final answer, and sets of *data D* of all natures. A key insight of the *Herbartian schema* is that the *milieu* is developed within a didactic system that includes both students (X) and teachers (Y), implying the collective development of *milieu* in the inquiry.

**Fig. 1:**
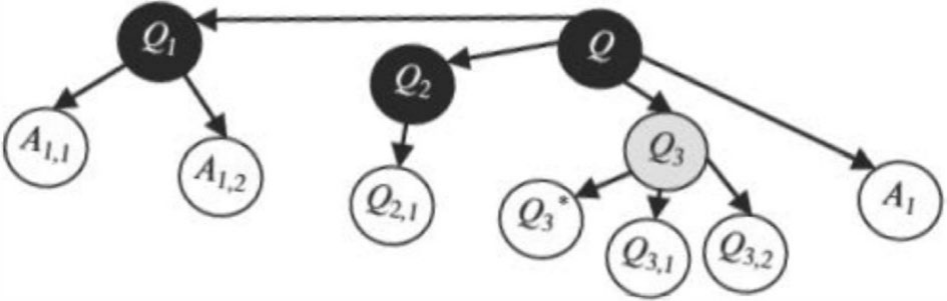
Example of Q–A map (Winsløw et al., 2013, p. 271)

Beyond the *Herbartian schema* that describes the principal objects involved in the inquiry, a tool called the *Question–Answer map* (Q–A map) is proposed to describe and analyze the dynamic process of inquiry (Winsløw et al., 2013). This tool allows us to consider the epistemological dimension of the inquiry and analyze how the mathematical knowledge at stake develops in the dialectic of questions and answers (Florensa et al., 2021). An example of a Q–A map is given in Fig. 1. We will adopt this analytical tool to trace the evolution of the *milieu* identified in the empirical data, highlighting the collective development of mathematical knowledge in Japanese lessons.

The concept of *milieu* is considered in relation to both an institution (the whole class) and its temporality (the lesson) in ATD. The epistemological dimension of the collective teaching is characterized by the evolution of the *milieu*, with which students and teachers are expected to interact throughout the lesson. The *milieu* in a lesson consists specifically of questions (including initial questions, subquestions, and derived questions), their answers (obtained from the media or elaborated in the classroom), related works, and data. It evolves based on classroom interactions and students’ individual and/or collective work. Thus, the *milieu* is co-constructed by students and teachers in the classroom, as the didactic system *S*(*X*; *Y*; *Q*) produces a *milieu M* in the Herbartian schema.

The progression of teaching and learning in the lesson constitutes a characteristic of the lesson, often described by Japanese lessons as a “drama.” To capture this aspect, we propose describing *milieus* according to the mathematical activity at a given moment, which is determined by a question or a set of questions linked to a specific mathematical content or students’ work. These *milieus* enable us to track their successive evolution. As mathematical activity shifts during teaching and learning, each *milieu* represents the epistemological dimension of a given moment. Here, the term *moment* is not used with exactly the same meanings as *didactic moments* in ATD (Chevallard & Bosch, 2019), but is instead associated with a specific mathematical activity.

Furthermore, in ATD, the development of mathematical knowledge is analyzed through three aspects that characterize the nature of mathematics teaching and learning: *topogenesis*, *mesogenesis*, and *chronogenesis* (Chevallard, 1991; Sensevy et al., 2005)^1^. From this perspective, the evolution of the *milieu* can be examined through *mesogenesis*, which includes the incorporation of new information and partial answers and their transformation into new ready-to-use knowledge tools to proceed with the inquiry (Sensevy et al., 2005). The distribution of responsibility between teachers and students in the progression of knowledge is addressed through *topogenesis* (Sensevy et al., 2005). The evolution of the knowledge proposed by the teacher and studied by the students over a single lesson or multiple lessons is considered through *chronogenesis* (Sensevy et al., 2005*).* These concepts provide a framework for analyzing the distinctive characteristics of Japanese lessons.

## *Milieu* as an analytical tool: illustration using Japanese lessons

The objective of this section is to illustrate how the concept of *milieu* can be used as an analytical tool for studying mathematics lessons. In the following, we first present the context of the Japanese lessons analyzed and then provide the results of the analysis for some specific phases of a lesson.

### Context: lessons about length

The lessons analyzed in this paper took place in a Grade 3 class (students aged 8–9) at a primary school attached to a Japanese university for teacher education. The teacher, referred to as Kazu (a pseudonym), has 12 years of teaching experience and specializes in mathematics. This unit consists of 15 lessons^2^ focusing on the measurement of length, particularly long lengths. According to the textbook, the learning objectives of this unit are to discover different measurement tools, to plan and experiment with measuring methods, and to define a unit of measurement. The first five lessons center on a single task: measuring the length of a school corridor. The subsequent ten lessons expand to measuring other lengths, including another corridor in the school, the perimeter of a sports field, a 1-km walk, and the distance between the school and a candy shop. For this paper, we analyze data from classroom video recordings and written student work collected during the first five lessons devoted to the first task, particularly Lessons 1 and 3 (Batteau, 2019; Batteau & Miyakawa, 2020). See Table 1 for an overview of these lessons.

**Table 1:** Description of the first five lessons

Lesson	Times (minutes)	Description of the lessons
1	32	Introduction of the instructional unit on length, which is named *feeling the lengths*
2	60	Students measure a long length (corridor) using the method, tool, and unit of measurement that were planned and selected during Lesson 1
3	65	Students share their results in the classroom and measure the length of the corridor with the measuring wheel to obtain accurate measurements and validate their results The class discusses students’ results, procedures, and, in particular, different units of measurement
4	100	The class summarizes the mathematical formula for measuring a length (length of units multiplied by the number of units is equaled to the entire length) Students measure the same corridor using other measuring tools
5	35	Whole-class discussion of students’ results and procedures

### Overall description and structure of lesson 1

**Fig. 2:**
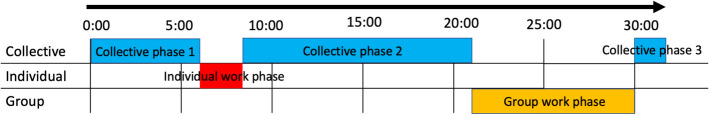
Structure of the lesson

The Lesson 1 consisted of whole-class discussions, as well as individual and group work. The structure of the lesson, in terms of time allocation and activity mode (collective, individual, and group), is shown in Fig. 2.

The lesson began with a whole-class activity involving the teacher and students (collective phase 1, 0:00–6:27), during which the teacher introduced the problem of the day and students shared their initial thoughts. This phase corresponds to the *hatsumon* phase in a Japanese problem-solving lesson. The main problem introduced was how to measure the length of the corridor, and the students provided some estimations, a process known as *mitōshi* (estimation).

Following this collective phase, the teacher asked students to justify their estimation, prompting a brief period of individual work (individual work phase, 6:27–7:45). During this time, students individually explored the reasons for their estimation, while the teacher walked around the classroom and occasionally interacted with them. This phase resembles *kikan-shidō*, as the teacher monitored students’ work and provided support as needed.

After this individual phase, the class returned to a whole-class discussion, which lasted more than ten minutes (collective phase 2, 7:45–21:20), on different measurement tools and methods to be used in the next lesson. This collective phase resembles *neriage*, though the class did not necessarily elaborate answers to the main problem but instead explored various possible methods.

Next, the class moved to group work for almost eight minutes (group work phase, 21:20–30:00) to decide which tools and methods they would use in the next lesson. Their discussions were based on the ideas explored in the previous collective phases.

The lesson concluded with a short summary (collective phase 3, 30:00–32:00).

The final state of the blackboard at the end of the lesson is shown in Appendix 3, providing additional insight into the overall organization of the lesson.

### Method of analysis

To identify the *milieu* that evolves during the lesson, we examined students’ and teachers’ questions and answers, as well as the works introduced accordingly to construct an answer to the question. Thus, in our analysis, each *milieu* consisted of questions (Q_i_), answers (A_i,j_)_,_ and works (W_k_).

A change in the *milieu* was identified by a shift in students’ mode of work or by a teacher’s question to alter students’ activities. All questions posed by either students or the teacher were considered, provided they pertained to mathematical content or students’ activities.

The *works* are objects that enable students to understand and analyze given answers and to construct their own answers. These *works* include not only mathematical objects or properties but also any materials or objects provided in the classroom to support students’ activities.

### *Milieus* in lesson 1

The lesson was collectively constructed with a storyline. In the following, we describe this process in terms of the evolution of *milieus*. As a result, we modeled the lesson using eight *milieus:* the *milieus* M_1_, M_2_, and M_3_ correspond to collective discussions during collective phase 1. The *milieu* M_4_ corresponds to the individual work phase. M_5_, M_6_, M_7_, and M_8_ correspond to collective phase 2. Not all *milieus* could be identified due to limited data, as the available video recordings do not contain sufficient footage of individual and group activities. The elements of the identified *milieus* are listed in Appendix 2.

#### Collective phase 1

**Fig. 3:**
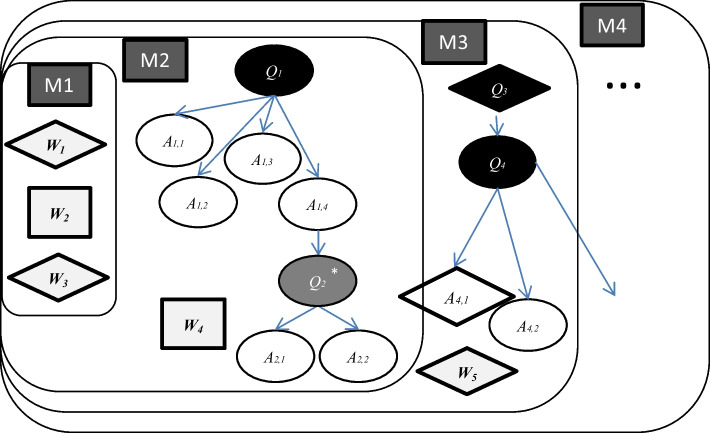
Presentation of the first three *milieus* during collective phase 1

This phase corresponds to the *hatsumon* phase, during which we identified three evolving *milieus* (M_1_, M_2_, and M_3_) layered on top of each other, as shown in Fig. 3 (see Appendix 1 for explanatory notes of the symbols used in this diagram).

*Milieu* M_1_ consists of three works introduced by the teacher at the beginning of the lesson: the title of the unit “feeling the length” (W_1_) written on the blackboard, “compass and ruler” (W_2_), and “the long length” (W_3_) written on the blackboard. These elements provide background information to help students better understand the question introduced by the teacher. This marks the beginning of the lesson’s storyline or drama.

*Milieu* M_2_ describes the initial development of the storyline through the first questions and answers: the teacher (Kazu) asks “What length did you measure in school last year?” (Q_1_). After some students’ answers, especially the one about the height of the school, which had already been measured (A_1,4_), this interaction leads to a derived question: “How tall was the school?” (Q^*^_2_). In this context, a new work “unit meter” (W_4_) is introduced and discussed.

M_2_ highlights the dynamic interactions between the teacher and students. The first question (Q_1_) elicits four student answers, while the derived question (Q^*^_2_) prompts two additional answers. The collective discussion provides the teacher with insights into students’ previous experiences with measurement and their areas of interest.

*Milieu* M_3_ includes the main question posed by the teacher: “How long is the length of the corridor in the school?” (Q_3_), along with a subquestion about estimating the corridor’s length (Q_4_). Although Q_3_ was explicitly asked by the teacher, it is considered a collectively constructed question, as it builds on the previous *milieus* (M_1_ and M_2_), particularly incorporating students’ interests.

The subquestion on estimation (Q_4_) arises from Q_3_ and elicits two student answers: “50 m” (A_4,1_ written on the board) and “I don’t think it’s 50 m” (A_4,2_). To support this estimation, Kazu referenced a familiar comparison length—the 50-m running track from the school’s sports festival (W_5_), which students already knew from everyday experience.

This analysis of *milieu*s demonstrates that the introduction of the problem (*hatsumon*) involves not only the teacher’s presentation of the problem but also a collective construction of the problem and an exploratory discussion about measurement estimation, grounded in students’ prior knowledge and experience.

#### Individual work phase

**Fig. 4:**
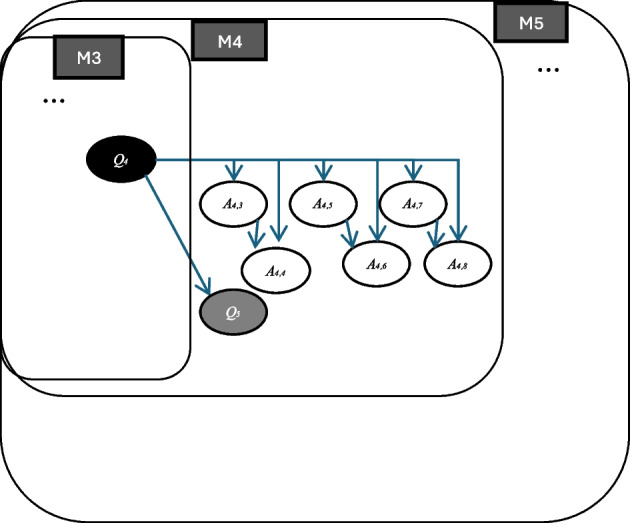
Presentation of the *milieu* M_4_ during the kikan-shidō phase

The next phase consists of a brief period of individual student work, which can be considered the *kikan-shidō* phase. The *milieu* M_4_ (Fig. 4) is characterized by individual student interactions during this phase. However, the collected data provides limited information on how the students interacted with the *milieu* during this phase.

*Milieu* M_4_, identified through students’ discussions, consists of answers to the subquestion Q_4_ from the previous *milieu* M_3_ concerning estimation and the new subquestion Q_5_ (“What is the reason for your estimation?”), which was posed by the teacher to several students. Since this *milieu* was reserved for several students, it did not evolve collectively within the whole class. However, it influenced the development of the subsequent *milieus*, as the subquestion Q_5_ was later discussed in the next collective phase.

#### Collective phase 2

**Fig. 5:**
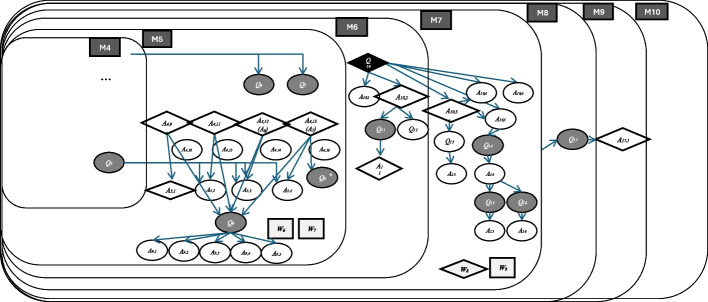
Presentation of the last *milieus* M_5_ to M_8_ (collective phase 2), the *milieu* M_9_ (group work phase), and the *milieu* M_10_ (collective phase 3)

Collective phase 2, which likely corresponds to the *neriage* phase, takes place after students’ individual work. We identified four *milieus* (M_5_ to M_8_) during this phase (see Fig. 5).

*Milieus* M_4_ and M_5_ are considered a twisted point in terms of the lesson as a “drama,” as they stem from two opposite answers (A_4,1_ and A_4,2_) and knowledge drawn from everyday experience (W_5_). *Milieu* M_5_ describes the collective development of the discussion, where students present estimation answers (A_4,9_, A_4,11_ A_4,13_, A_4,15_) alongside objections (A_4,10_, A_4,12_ A_4,14_, A_4,16_). Students also provide justifications for their estimations (A_5,1_, A_5,2_, A_5,3_). For example, in A_5,1_, a student’s height is used as a work (W_6_): a third grader is approximately 1 m tall; since the corridor’s length is equal to the collective length of 35 lying students (the entire class), it is estimated to be around 35 m. The discussion further evolves with three additional subquestions (Q_6,_ Q_7,_ Q_9_) introduced through student–teacher interactions. These questions guide students to place their estimations within specific length intervals: less than 35 m, between 35 and 60 m, between 60 and 125 m, between 125 m and 1–2 km (Q_9_). These subquestions facilitate the comparison of students’ answers, which should be a key objective of this collective phase.

Following the collective activity on measurement estimation modeled by M_5_, the subsequent activity, which constitutes the *milieu* M_6_, focuses on exploring the measuring methods and tools. In this activity, the teacher poses the subquestion “How can you measure the length of the corridor?” (Q_10_). Two answers emerge: using a measuring wheel (A_10,1_) and using a tape measure (A_10,2_). The subquestion “Why use a tape measure?” (Q_11_) then arises, particularly from the interaction around the answer A_10,2_. Additionally, a student’s subquestion “What is a tape measure?” (Q_12_) stems from this answer.

The *milieu* M_7_ models discussion on a measuring method proposed by a student as an answer to question (Q_10_). This method (A_10,3_) involves measuring the corridor by marking one meter with a one-meter object. Specifically, this student proposed to use a compass to measure one meter and then repeat the process. This answer triggers several questions and answers from other students. For example, a student asked the subquestion, “Do you write it (the mark) on the floor?” (Q_13_). Another student asked “Why don’t we need a lot of one-meter objects?” (Q_14_). From these interactions, result two subquestions “For example, how do you measure the length of this blackboard?” (Q_15_) and “There is only one [object of one meter], can we do it?” (Q_16_). These interactions during M_7_ illustrate how the students’ ideas collectively develop the measuring method.

The final *milieu* M_8_ includes a subquestion that comes from these discussions on measuring methods modeled by M_7_: “What do you do if there is no measuring wheel and tape measure for each group?” (Q_17_).

#### Group work phase

During this phase, groups of students discuss how to measure the corridor and decide on one method to be applied in the next lesson. The chosen measuring method must exclude the use of a measuring wheel or tape measure, as these will be used to verify the results at the end.

The activity in this phase can be modeled by different *milieus*, one of which is M_9_, although the available data is insufficient to identify all of them. Some groups of students discuss the elements written on the blackboard, while others focus on different measuring tools already presented during the collective phase.

A group of students we observed proposed the answer “With a big compass (the method is drawn on the blackboard) with the unit 16 cm 5 mm” (A_17,1_). This interaction demonstrates how students engage with the *milieu*, which incorporates elements from the *milieus* developed in the previous phases.

#### Collective phase 3

The final phase, considered the *matome* phase, is brief because the primary objective of this lesson was to introduce the measurement problem, which will be explored in subsequent lessons. The teacher only asks if students have chosen a measurement tool and method for measuring the corridor’s length. Although the teacher reformulates the question Q_17_, the *milieu* does not receive any new elements. The *milieu* M_10_ encompasses the *milieus* from previous phases.

### *Milieus* in lesson 3

The same method for analyzing the *milieu* was applied to Lesson 3. Here, we present only the results of this analysis, as depicted in Fig. 7. The blackboard at the end of the lesson given in Appendix 4 indicates the overall organization of this lesson. This lesson is modeled with 11 *milieus*. *Milieu* 1 is identified during the presentation of the measurement results of the corridor. *Milieu* 2 describes a comparison of these results to assess their accuracy. *Milieu* 3 relates to a discussion about the operations of a measuring wheel in preparation for the next activity: measuring the length of the corridor using the measuring wheel (*Milieu* 4). The discussion then shifts to addressing a technical issue with the measuring wheel (*Milieu* 5), followed by a discussion of the measurements obtained with this tool (*Milieu* 6). In smaller groups, students are asked to formulate the measuring methods used in Lesson 2 for measuring the corridor’s length (*Milieus* 7 and 8). After group work, the class shares these measuring methods on the blackboard (*Milieu* 9), leading to a discussion about the reasons behind variations in their measurements (*Milieu* 10). Finally, the class concludes by summarizing the tools used and the units for measurement (*Milieu* 11).

In *Milieus* 9, 10, and 11, we may find a Q–A map structure that differs slightly from other parts of the lessons, particularly from Lesson 1. These *milieus* include a sequence of questions, each typically followed by a single answer, indicating the teacher’s strong guidance toward the end of this lesson.

## Discussion

The discussion is organized around collective, chronological, and epistemological dimensions of Japanese lessons in terms of the three key aspects of the development of mathematical knowledge within ATD: *topogenesis*, *mesogenesis*, and *chronogenesis*.

### Collective dimension of the lesson

**Fig. 6:**
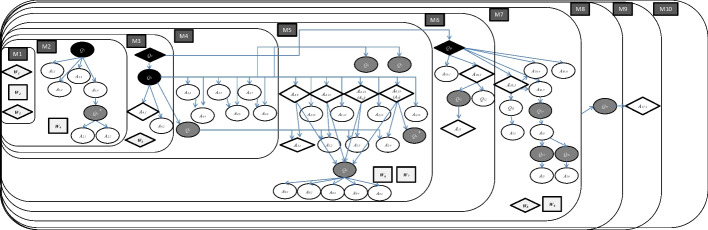
Nested *milieus* of Lesson 1

**Fig. 7:**
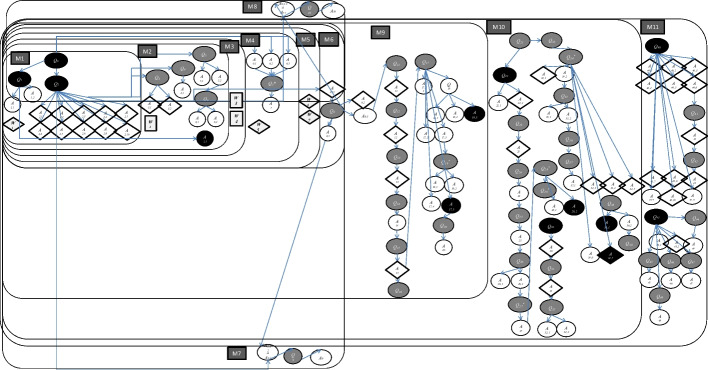
Successive *milieus* in Lesson 3

As previously stated, the collective dimension is one of the distinct characteristics of Japanese mathematics lessons. In the lessons analyzed in the previous section, this dimension could be observed not only during the collective phases of the Japanese lesson (Fig. 2) but also throughout other phases of the lesson. This dimension can first be explained in terms of *mesogenesis*, which concerns the evolution of the *milieu*. As shown in Figs. 6 and 7, the *milieu* developed through the contributions of both the teacher and students. Some elements emerged from students’ responses to the teacher’s questions during the collective phases, while others arose from individual or group work. These elements were then reintegrated into subsequent discussions and activities, reinforcing the collective learning process.

The collective development of *milieu* can be further deepened in terms of the *topogenesis*, which pertains to the roles or positions of participants in the construction of mathematical knowledge. The Q–A maps (Figs. 3, 4, 5, 6, and 7) illustrate this through the use of colors (black, grey, and white) to represent the respective *topos* of students and the teacher. In a typical mathematics lesson, students’ role is primarily to answer the questions, while the teacher asks questions. However, in Japanese lessons, a teacher’s role extends to formulating questions based on students’ responses allowing the class to progressively develop *milieus* throughout the learning process. This explains the high occurrence of grey questions (11 out of 17 for Lesson 1 and 39 out of 48 for Lesson 3), reflecting student-teacher interactions, and why many questions are derived from other *milieus*. Such a finding highlights students’ active roles in their learning process while the teacher manages the process of learning by incorporating their interests and responses.

Furthermore, students interact with the *milieu* in whole-class discussion as well as in group work, contributing to its continual evolution. The *milieu* can thus be considered according to different institutions, whether the entire class, a student group, or even an individual student.

### Chronological dimension

Another key characteristic of Japanese mathematics lessons is their chronological dimension, which shapes the flow of the lesson—also described as a “drama.” Our observations reveal two distinct levels of lesson flow: the micro level within a single lesson and the macro level across a series of lessons.

At the micro level, the chronological dimension is reflected in the structured problem-solving approach within a single lesson. For instance, in Lesson 1, the lesson unfolds through four phases: introduction (*hatsumon* phase), described by *milieus* M1–3; individual work (*kikan-shidō* phase), by *milieu* M4; collective discussion (*neriage* phase), by *milieus* M5–8; summary (*matome* phase), by *milieus* M9–10. This structured progression, described by the evolution of *milieus* (*mesogenesis*), ensures that mathematical knowledge emerges gradually and meaningfully within the lesson. The *chronogenesis* of mathematical knowledge is reflected in the sequential emergence of key elements of the *milieu* (questions, answers, and works), as vividly illustrated in Figs. 6 and 7.

At the macro level, the chronological dimension extends beyond individual lessons to the entire sequence of lessons. Table 1 outlines the classroom activities in the first five lessons, revealing a broader flow of mathematical knowledge development. One may again identify the progression of structured problem-solving lessons: Lesson 1 for the introduction of the main problem, Lesson 2 for group work to explore possible solutions, Lesson 3 for the development of mathematical ideas and methods (*neriage*), Lessons 4 and 5 for the summary of the mathematical formula for measuring a length, demonstrating that the length of a unit multiplied by the number of units equals the total length (*matome*). During Lesson 4, the teacher used students’ procedures discussed in Lesson 3 to decontextualize the formula in a general form. Then, he asked students to apply it with their data, leading to a recontextualization of the formula. The latter part of Lesson 4 involved measuring the corridor using different units and applying the formula. During Lesson 5, students extended their approach by measuring other long lengths within their school, reinforcing the measurement method introduced. This overarching progression also exemplifies the *chronogenesis* of mathematical knowledge, illustrating how concepts evolve over time within the sequence of lessons.

In these flows of lessons, the teacher plays a crucial role (*topos*) in managing the timing of when mathematical ideas (elements of *milieu*) are introduced, ensuring that knowledge emerges at appropriate moments. For example, in our observed lessons, measurement using a measuring wheel and tape measure is not allowed immediately but only after students first explore other methods for measuring the corridor’s length. This sequencing was intended to first find the formula for measuring a length.

This implies that the four phases of the structured problem-solving lessons serve as an instructional framework for managing the three key aspects of mathematics teaching: the evolution of the *milieu* (*mesogenesis*), the structured flow (*chronogenesis*), and the roles assigned to the teacher and students (*topogenesis*).

### Epistemological dimension of lesson

Another characteristic of the Japanese lesson is the place allocated to the knowledge in the lessons. Japanese teaching is linked to a specific goal of the lesson, which is the development and learning of new mathematical knowledge. The notion of *milieu* precisely captures this epistemological dimension along with the evolution of *milieus*. This dimension is especially related to the *mesogenesis*.

As explored in this paper, the analysis of *milieus* in Lessons 1 and 3 indicates that teaching measurement extends beyond the technicalities of tools and methods. It encompasses the understanding of measurement, emphasizing the importance of measurement estimation and the use of various tools to yield a variety of units, as evidenced by the blackboard in Appendix 4. Specifically, the classroom activities in Lesson 1 are enriched by different types of work that contribute to the conceptual understanding of measurement: mathematical objects (W_1_, feeling the length; W_3_, the long length; W_4_, the unit meter; W_7_, the unit kilometer), measuring tools (W_2_, compass and ruler; W_8_, one-meter object; W_9_, ruler of one meter), and ordinary life (W_5_, 50 m, the distance that first and second graders run during a sports festival; W_6_, the height of a student).

Notably, the epistemological dimension of Lesson 1 is echoed in previous research on the teaching and learning of measurement. The above-mentioned works were used in two methods of measurement estimation: comparison to a reference point and mental report of a referent (Hartono, 2015; Joram et al., 1998). The critical role of measurement estimation in understanding measurement, understanding the units of measure, and their interrelations has also been recognized (Joram et al., 1998; Sirieix, 2019). Additionally, Chambris and Batteau (2021) point out the importance of the unit concept and the necessity of obtaining a variety of units for the multiplicative reasoning involved in length measurement.

### Management of the blackboard

Through our analysis of Japanese mathematics lessons in this study, we find that the management of board writing plays a critical role in all three aspects of mathematical knowledge development: *topogenesis*, *mesogenesis*, and *chronogenesis*. In general, Japanese teachers place great importance on the organization of board writing, known as ‘*bansho*’ (Tan et al., 2018; Yoshida, 2005), which serves as a vital tool for facilitating collective problem-solving and is a distinctive characteristic of Japanese mathematics lessons. In elementary and lower secondary schools, effective board management is regarded as an essential teaching skill. The blackboard is carefully structured to visually represent the historical progression of classroom activities, students’ thinking processes, and the mathematical knowledge developed during the lesson. Because teachers typically avoid erasing any part of the blackboard during the lesson, the final state of the board serves as a comprehensive record of the lesson’s structure and organization. This emphasis on board management is reflected in the online community, particularly the Facebook group “Bansho Book,” which has over 7000 participants who share images of blackboards and engage in discussions on mathematics teaching practices.

In the lessons we analyzed, the elements written on the blackboard constitute the *milieus* for the entire lesson, providing students with ongoing reference throughout. The Japanese approach of blackboard use enables a continuous evolution of *milieus* in a nested manner, illustrating the *mesogenesis* of mathematical knowledge. The collective dimension of Japanese lessons further highlights the critical role of the blackboard as an official space for organizing and displaying the elements of *milieus* (see Appendixes 3 and 4). Additionally, the chronological dimension, corresponding to *chronogenesis*, is evident in how board writing visually represents the flow of the lesson, reinforcing the progressive development of mathematical knowledge in the classroom.

Regarding *topogenesis*, it is the teacher’s role to organize the blackboard, while students contribute to the *milieu* by providing elements of *milieu* (answers, questions, and works). The teacher, in turn, evaluates these contributions, either rejecting or integrating them into the *milieu* for further use. In our study, the teacher strategically incorporated key mathematical elements, such as the concept of unit (Isoda & Katagiri, 2012), measurement methods, tools, units of measurement, and estimations of measurement.

## Conclusions and perspectives

This paper presents an analysis of Japanese problem-solving lessons using the theoretical tool of *milieu* from the perspective of the Anthropological Theory of the Didactic (ATD). This analytical tool has been adapted to deepen the distinctive characteristics of Japanese problem-solving lessons, particularly focusing on the three dimensions discussed in the preceding section. By examining the evolution of *milieus*, this approach highlights how mathematical knowledge dynamically develops throughout the lesson.

Beyond Japanese lessons, we consider that this method of analysis has broader applicability for analyzing mathematics lessons in diverse educational contexts. Future research will involve applying this analytical tool to lessons in different settings, ideally as part of an international comparative study.

Additionally, while our analyses provide valuable insights, they do not fully capture the interplay between individual learning and collective dimension of the lesson, due to limited data on individual and group activities. Further investigation of this aspect could shed light on how individual learning happens within collective teaching, offering further insights into the specificity of Japanese problem-solving lessons.

## Figures and Tables

**Table 2:**
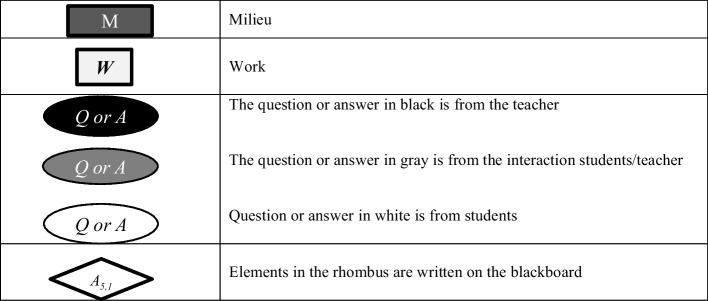
Explanatory notes of symbols used in the diagram of *milieus*

## Appendix 1

Table 2

## Appendix 2

Table 3

**Table 3:** List of questions, answers, and works by *milieus* for Lesson 1

M_1_	W_1_: Feeling the length (written on the blackboard) W_2_: Compass and ruler W_3_ The long length
M_2_	Q_1_: What length did you measure in school last year? A_1,1_: The length of the wall bars in the gym A_1,2_: The length of the frame displayed at the entrance A_1,3_: The length of the stairs leading to the rooftop A_1,4_: School height Q_2_*: How tall was the school? A_2,1_: I don’t know A_2,2_: About two thousand meters W_4_: Unit meter
M_3_	Q_3_: How long is the length of the corridor in the school? (written on the blackboard) Q_4_: How long do you think is the length of the corridor? A_4,1_: About 50 m (written on the blackboard) A_4,2_: I don’t think it’s 50 m W_5_: 50 m is the length that first and second graders run during a sports festival (written on the blackboard)
M_4_	A_4,3_: About 80 m A_4,4_: About 50 to 80 m A_4,5_: 8 m A_4,6_: This is 1 m, so 8 m is strange A_4,7_: 30 m A_4,8_: For 30 m, there is no reason Q_5_: What is the reason for your estimation?
M_5_	A_4,9_: About 35 m (written on the blackboard) A_5,1_: The third-grade student’s height is about 1 m, and I think it will be the length of the corridor when everyone lies on the floor (written on the blackboard) W_6_: height of a student A_4,10_: I don’t think it’s 35 m A_4,11_: 60 m (written on the blackboard) A_4,12_: I don’t think it’s 60 m A_5,2_: Intuition Q_6_: Is there anyone who thinks it is longer than 60 m? A_4,13_ (A_6_): 125 m (written on the blackboard) A_4,14_: 125 m is strange A_5,3_: Intuition Q_7_: Is there anyone who thinks it is longer than 125 m? A_4,15_ (A_7_): 1 or 2 km (written on the blackboard) Q_8_: Do you know the kilometer? W_7_: Unit kilometer A_5,4_: Intuition A_4,16_: I can’t think it’s 1 or 2 km Q_9_: Where does the length that you estimated belong: less than 35, between 35 and 60, between 60 and 125, between 125 m and 1 or 2 km? A_9,1_: less than 35 (students answer with their hand up) A_9,2_: between 35 and 60 (students answer with their hand up) A_9,3_: between 60 and 125 (students answer with their hand up) A_9,4_: between 125 m and 1 km (students answer with their hand up) A_9,5_: 1 or 2 km (students answer with their hand up)
M_6_	Q_10_: How do you measure the corridor? (written on the blackboard) A_10,1_: Using a “measuring wheel” (drawn on the blackboard) A_10,2_: Using a tape measure (written on the blackboard) Q_11_: Why use a tape measure? A_11_: It can measure any length (written on the blackboard) Q_12_: What is a tape measure?
M_7_	A_10,3_: Using an object of one meter, such as a compass, measure the corridor by marking one meter and repeating it (written and drawn on the blackboard) W_8_: Object of 1 m (written on the blackboard) Q_13_: Do you write it (the mark) on the floor? A_13_: I do not mark it with a pencil A_10,4_: I must find a perfect object of one meter. (So it’s impossible) A_10,5_: There are not enough objects of one meter in the school. (So it’s impossible) Q_14_: Why don’t we need a lot of objects of one meter? A_14_: I mark the end of one meter and bring the other end of the one meter to the mark. And again, I mark the end. I repeat it Q_15_: How do you measure the length of this blackboard? Q_16_: There is only one, can we do it? W_9_: Ruler of one meter A_16_: Yes, we can A_15_: (The teacher and one student measure the length of the blackboard) A_10,6_: I measure using something like an open compass. I measure the half-finished parts with another thing
M_8_	Q_17_: How do you do without using “measuring wheel” and tape measure?
M_9_	Group work: “Group n°… will measure with….” (Sentence written on the blackboard, that student must complete) A_17,1_: with a big compass (the method is drawn on the blackboard) with the unit 16 cm 5 mm
M_10_	

## Footnotes

1 The notions of *topogenesis* and *chronogenesis* are primarily proposed in the Theory of Didactic Transposition (Chevallard, 1991).

2 This unit consists of 22 periods of 30 min, which are called *modules* in this school. Each lesson consists of one or two *modules*.
